# Analysis of aortic blood-flow from ECG-free realtime PC MRI

**DOI:** 10.1186/1532-429X-17-S1-Q42

**Published:** 2015-02-03

**Authors:** Markus Huellebrand, Anja Hennemuth, Lennart Tautz, Arun Joseph, Jens Frahm

**Affiliations:** 1 Fraunhofer MEVIS, Bremen, Germany; 2 Max Planck Institute for Biophysical Chemistry, Goettingen, Germany

## Background

Realtime PC MRI flow image data is usually acquired in combination with ECG signal acquisition, which enables the separation of the image sequences into cardiac cycles. Thereby, it becomes possible to analyze the variation of bloodflow-related parameters through influences such as arrhythmia or breathing maneuvers.

Because of the time consuming ECG lead positioning and the errors occurring through interferences with MRI gradients, it is desirable to be able to do the cycle separation without the ECG signal for sequences which do not require ECG triggering.

Our approach for an ECG-free multicycle flow analysis is based on the image data only. In order to compare the image-based cycle separation with the ECG-based approach, the suggested method has been applied to data acquired with ECG-signal, and the differences in cycle separation as well as their influence on the clinically relevant parameters have been analyzed.

## Methods

20 data sets of young subjects (average age 25 years) were acquired with a 3-T MR system (TrioTim, Siemens, Erlangen, Germany). A phase encoded under-sampled radial FLASH sequence that is reconstructed with a regularized nonlinear inversion, was used for the flow analysis of the ascending aorta (spatial resolution= 1.33x1.33x6.0mm^3^, temporal-resolution=42ms, VENC=200cm/s, flip-angle=10°). Images were acquired continuously every 42 ms also recording the trigger time, provided by the scanner.

Data were analyzed with the research software CAIPI extending the existing approach by an automatic cycle detection based on the analysis of the blood flow curves.

To this end a static region inside the vessel of interest is detected automatically for which the flow curve of the whole sequence consisting of 369 time-points is calculated. Analyzing these curves, we detect the time frames closest to the r-peak. The additional information are then provided to the automatic vessel contour propagation to all time frames, after a manual initialization of the aortic vessel wall in one time-frame [Huellebrand et al.: "Automatic quantification of blood-flow from real-time phase-contrast mri." in RSNA (2013)].

## Results

For method comparison, the quantitative analysis for a user-defined segmentation of the ascending aorta has been performed based on the ECG-based cycle separation as well as for the image-based cycle separation.

Incomplete cycles at the beginning and the end of the sequence were excluded in both cases.

For the complete cycles the difference of the start index per cycles is calculated.

Table 1 shows the statistics of the error per subject. The influence on the clinical parameters is shown in Table 2.

**Table 1:** Cardiac cycle start index comparison

Subject id	Minimum	5% Percentile	25% Percentile	Median	75% Percentile	95% Percentile	Maximum
1	-3	-1.7	0	0	0	1.35	2

2	-2	-0.6	0	0	0	1	1

3	-1	-1	0	0	0	1	1

4	-1	-1	0	0	0	1	1

5	0	0	0	0	0	0	0

6	0	0	0	0	0.25	1	1

7	-2	-2	-1	-1	0	0.15	1

8	-1	-1	0	0	0	0	0

9	-3	-2.05	0	0	0	0.05	1

10	-1	-1	-1	0	0	1	1

11	-3	-1.5	-1	0	0	1	1

12	-2	-2	-1	0	0	1	1

13	-1	-1	0	0	0.25	1	1

14	-1	-1	0	0	1	1	1

15	-1	-0.3	0	0	0	1	1

16	-1	-1	-0.25	0	1	1	1

17	-1	-1	0	0	1	1	1

18	0	0	1	1	1	2	2

19	0	0	0	1	1	1	1

20	0	0	1	1	1	1.3	2

The table shows the statistics of difference of the start indices for the cardiac cycles per subject.

**Table 2:** Clinical parameters shows the difference of flow, peak velocity, regurgitant fraction and heart rate between the proposed method (A) and the reference approache (B).

Comparison		Flow [ml]	Flow [l/min]	Peak Velocity [cm/s]	Regurgitant Fraction	Heart Rate [bpm]
Param_A_-Param_B_	Mean	0.02	-0.03	0.01	0.0	-0.26
	
	Standard deviation	1.01	0.29	0.16	0.15	2.83

|Param_A_-Param_B_|	Mean	0.6	0.19	0.01	0.06	1.86
	
	Standard deviation	0.4	0.08	0.05	0.09	0.87

max(|Param_A_-Param_B_|)	Mean	1.92	0.56	0.11	0.22	5.39
	
	Standard deviation	1.34	0.27	0.48	0.25	3.01

## Conclusions

The comparison of the quantification with and without ECG information shows a good agreement for the clinical parameters of the aortic blood flow. Further studies with different patient populations and different vascular regions will evaluate, if an ECG-free realtime acquisition has the potential to become a standard acquisition technique in clinical practice.

## Funding

Fraunhofer and Max-Planck Society.

